# Host-pathogen coevolution drives innate immune response to *Aphanomyces astaci* infection in freshwater crayfish: transcriptomic evidence

**DOI:** 10.1186/s12864-022-08571-z

**Published:** 2022-08-22

**Authors:** Ljudevit Luka Boštjančić, Caterina Francesconi, Christelle Rutz, Lucien Hoffbeck, Laetitia Poidevin, Arnaud Kress, Japo Jussila, Jenny Makkonen, Barbara Feldmeyer, Miklós Bálint, Klaus Schwenk, Odile Lecompte, Kathrin Theissinger

**Affiliations:** 1 LOEWE Centre for Translational Biodiversity Genomics (LOEWE-TBG), Senckenberg Biodiversity and Climate Research Centre, Georg-Voigt-Str. 14-16, 60325 Frankfurt am Main, Germany; 2 Institute for Environmental Sciences, University of Koblenz-Landau, Fortstrasse 7, 76829 Landau, Germany; 3 Department of Computer Science, ICube, UMR 7357, University of Strasbourg, CNRS, Centre de Recherche en Biomédecine de Strasbourg, Rue Eugène Boeckel 1, 67000 Strasbourg, France; 4 Department of Environmental and Biological Sciences, University of Eastern Finland, P.O. Box 1627, 70210 Kuopio, Finland; 5 Present address: BioSafe - Biological Safety Solutions, Microkatu 1, 70210 Kuopio, Finland

**Keywords:** Crayfish plague, *Astacus astacus*, Invertebrate immune mechanisms, Innate immune system, *Procambarus virginalis*, Differential gene expression, de novo assembly, Hepatopancreas, Novel immune-related genes

## Abstract

**Background:**

For over a century, scientists have studied host-pathogen interactions between the crayfish plague disease agent *Aphanomyces astaci* and freshwater crayfish. It has been hypothesised that North American crayfish hosts are disease-resistant due to the long-lasting coevolution with the pathogen. Similarly, the increasing number of latent infections reported in the historically sensitive European crayfish hosts seems to indicate that similar coevolutionary processes are occurring between European crayfish and *A. astaci*. Our current understanding of these host-pathogen interactions is largely focused on the innate immunity processes in the crayfish haemolymph and cuticle, but the molecular basis of the observed disease-resistance and susceptibility remain unclear. To understand how coevolution is shaping the host’s molecular response to the pathogen, susceptible native European noble crayfish and invasive disease-resistant marbled crayfish were challenged with two *A. astaci* strains of different origin: a haplogroup A strain (introduced to Europe at least 50 years ago, low virulence) and a haplogroup B strain (signal crayfish in lake Tahoe, USA, high virulence). Here, we compare the gene expression profiles of the hepatopancreas, an integrated organ of crayfish immunity and metabolism.

**Results:**

We characterised several novel innate immune-related gene groups in both crayfish species. Across all challenge groups, we detected 412 differentially expressed genes (DEGs) in the noble crayfish, and 257 DEGs in the marbled crayfish. In the noble crayfish, a clear immune response was detected to the haplogroup B strain, but not to the haplogroup A strain. In contrast, in the marbled crayfish we detected an immune response to the haplogroup A strain, but not to the haplogroup B strain.

**Conclusions:**

We highlight the hepatopancreas as an important hub for the synthesis of immune molecules in the response to *A. astaci*. A clear distinction between the innate immune response in the marbled crayfish and the noble crayfish is the capability of the marbled crayfish to mobilise a higher variety of innate immune response effectors. With this study we outline that the type and strength of the host immune response to the pathogen is strongly influenced by the coevolutionary history of the crayfish with specific *A. astaci* strains*.*

**Supplementary Information:**

The online version contains supplementary material available at 10.1186/s12864-022-08571-z.

## Background

Host-pathogen interactions are models for evolutionary arms-races, thus cycles of reciprocal co-adaptation [1]. Coevolution between hosts and pathogens is ubiquitous, often resulting in rapid evolutionary change, and is linked to the maintenance of diversity [2, 3]. Pathogens impose strong selection on their hosts which try to minimize their fitness loss, e.g. by evolving resistance, while pathogens themselves are under strong selection to undermine host defences without causing the complete collapse of the host population [4]. Parasite virulence may peak after a host-jump, as the new host has not yet evolved any specific defence mechanisms [4, 5]. The theory behind host-parasite interactions is well established [6, 7], and there are ample examples for coevolutionary adaptations [8, 9]. However, we are only just starting to understand the underlying genomic mechanisms and genes involved in co-adaptation processes [10]. Host-pathogen interactions are of high interest in conservation biology, as they not only determine the fate of invasive species, but they also affect the survival of native taxa [11]. Due to its high importance for aquaculture and management, scientists have studied the interaction between freshwater crayfish and their pathogen *Aphanomyces astaci* for over a century [12]. Still, the coevolutionary aspect of this host-pathogen interaction remains understudied.

Likely because of their coevolutionary history, North American crayfish species are generally considered resistant to the pathogen *A. astaci,* the causative agent of crayfish plague disease [13, 14]. It is assumed that these crayfish species are natural carriers of their specific *A. astaci* strain, usually efficiently preventing it from spreading inside their tissues through melanisation mediated encapsulation of the pathogen hyphae in the cuticle [15, 16]. In contrast, European crayfish species do not naturally carry the pathogen and are considered susceptible to the disease [17–19]. Therefore, the introduction of invasive North American crayfish species into Europe, and with them of *A. astaci*, caused mass mortalities and local extinctions among European crayfish populations [20]. The *A. astaci* strains present in Europe can be grouped into 4 different haplogroups [21]. Haplogroup A contains strains of unequal virulence (ranging from non-virulent to highly virulent), while haplogroups B, D and E are usually characterized by high virulence [17, 22, 23]. Despite the high susceptibility of native European crayfish species towards the crayfish plague disease agent, latent crayfish plague infections without mass mortalities have been reported for several species infected with low virulent *A. astaci* strains of haplogroup A [12], suggesting the presence of an ongoing dynamic coevolutionary process. However, the foundation of this naturally occurring resistance to *A. astaci* remains unclear.

Initial studies suggested that one of the main factors contributing to the resistance of North American crayfish species is the constitutively over-expressed prophenoloxidase (proPO) in the haemocytes, a key enzyme in the encapsulation of pathogens in melanin [24]. Conversely, in European crayfish species, the expression of this enzyme is dependent on stimuli of the pathogen [24]. Based on the current knowledge of the innate immunity mechanisms in crustaceans, the response to pathogens comprises both cellular and humoral components, with the proPO cascade playing part in the humoral response [25–27]. The immune response is triggered by the pathogen-associated molecular patterns (PAMPs), such as β-(1,3)-glucan, which is one of the main constituents of the oomycetes cell wall [28]. These molecules are recognised by specific pattern-recognition proteins (PRPs) of the host, which can exist as soluble molecules or as associated with cell membranes. PRPs of particular relevance are lectin-like proteins, Down Syndrome Cell Adhesion Molecules (DSCAMs) and Toll-like receptors (TLRs) [25, 29]. The interaction between ligands and receptors leads to the activation of different molecular pathways involved in the humoral or cellular response, all of them coordinated by the core mediators of the crustacean immunity, the haemocytes. Haemocytes are crucial for the processes of phagocytosis, encapsulation and melanisation, and they are involved in delivering the molecular effectors of the humoral response, such as antimicrobial peptides and proPO, to the infection sites [27, 30, 31].

The mechanisms underlying the crayfish immune response to *A. astaci*, however, is much more complex than the simple activation of the proPO cascade, but its molecular effectors and other tissues beyond haemolymph have not received much attention. In Crustaceans, hepatopancreas represents an integrated organ of immunity and metabolism [32, 33]. It plays a major role in pathogen clearance, antigen processing [34, 35], detoxification, and heavy metal deposition [36]. It also serves as a source for immune molecules, which can be released from the epithelial cells into the haemocoel sinusoids, allowing for their rapid distribution in the haemolymph of the crayfish [33]. In recent years, the involvement of the hepatopancreas in the response to various disease and environmental factors has been highlighted in crustaceans [36–40]. However, its role in the immune response to *A. astaci* infection has not been clearly defined.

Through the coevolutionary transcriptomics approach, we aimed to deepen our understanding of the molecular mechanisms underlying the resistance and susceptibility of freshwater crayfish to the *A. astaci*, to unravel how coevolution is shaping the molecular response to the pathogen. By analysing gene expression profiles in the hepatopancreas, we compared the immune response of the susceptible native European noble crayfish (*Astacus astacus*) and the resistant invasive marbled crayfish (*Procambarus virginalis*) to an *A. astaci* challenge. In a controlled infection experiment, both species were infected with a highly virulent (haplogroup B, hereinafter Hap B) and a lowly virulent (haplogroup A, hereinafter Hap A) *A. astaci* strain [41]. Previous studies focused on the early stages of the *A. astaci* infection, but the transition from acute infection to latent infection states has not been studied. Therefore, the hepatopancreas of the crayfish was sampled during the early phase of challenge (day 3) and late phase of the challenge (day 21).

We hypothesised that the hepatopancreas is a highly relevant tissue in the immune response towards *A. astaci* infections, and we expected to detect several immune-related transcripts in all treatment groups. We expected that the gene expression profiles of the immune-related transcripts differ between the noble crayfish and the marbled crayfish, reflecting the species’ different coevolutionary history with the specific *A. astaci* strain, and thus their different abilities to defend against the pathogen. Furthermore, for the susceptible noble crayfish, we expected a stronger immune response in noble crayfish challenged with the highly virulent Hap B strain compared to the less virulent Hap A strain. Conversely, we did not expect any gene expression difference among treatment groups for the resistant marbled crayfish. Lastly, we expected the latently infected crayfish to show a chronic immune response against *A. astaci,* with the presence of differentially expressed immune-related transcripts 21 days post-challenge.

The results presented in this paper deliver novel insights into the gene repertoire involved in the immune response to the *A. astaci* challenge, deepening our understanding of freshwater crayfish immunity and their interaction with the pathogen, *A. astaci.*

## Results and discussion

### Immune-related transcripts in the hepatopancreas, the mediator of the crayfish immune response to *A. astaci* challenge

**Fig. 1:**
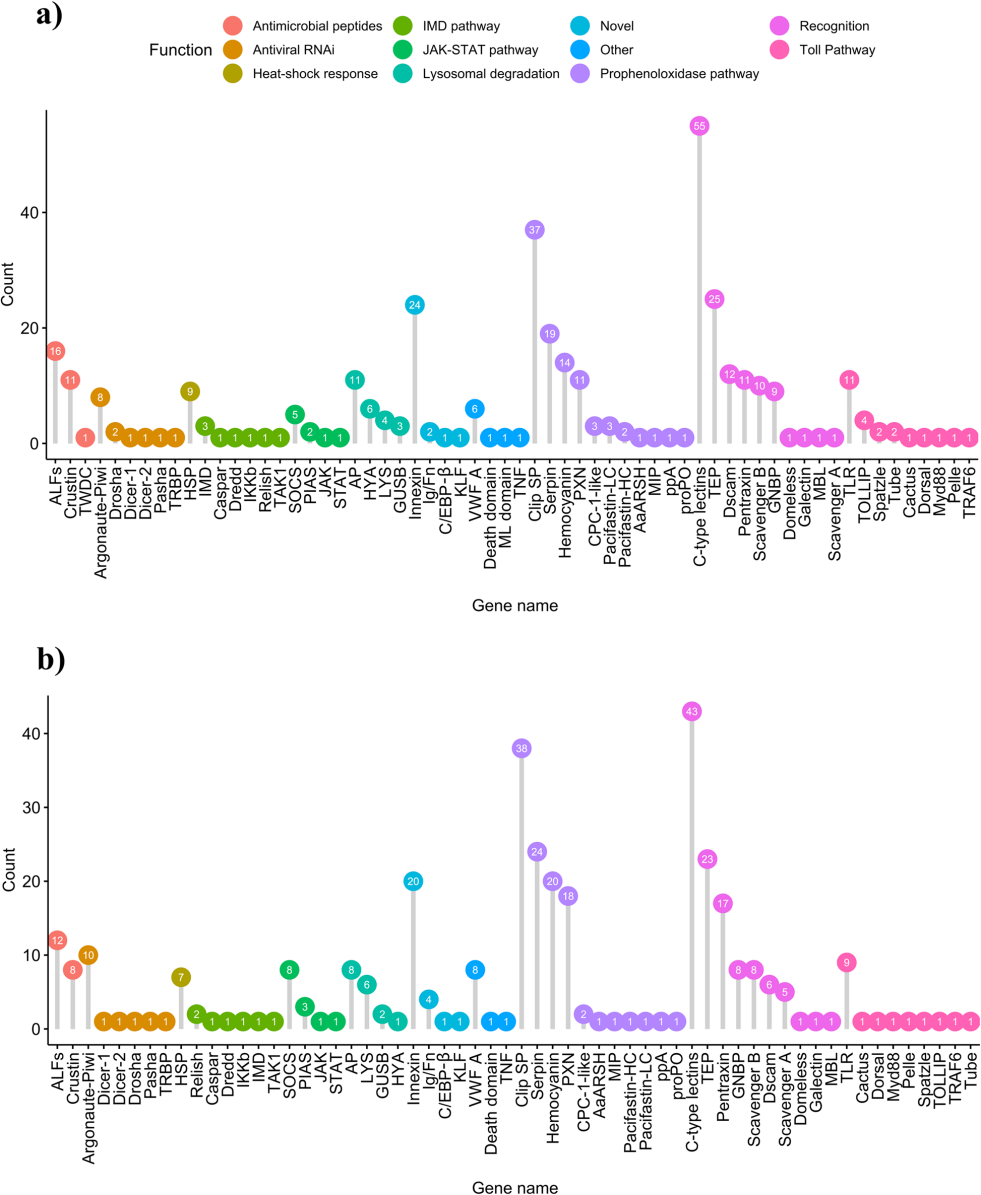
Genes involved in the representative immune related pathways, identified thought the similarity-based approach in (**a**) the noble crayfish and (**b**) the marbled crayfish. For all genes abbreviations are available in the Table S7

Genomic research on non-model organisms is faced by the challenge of annotating large sets of genes from unknown origin. This challenge is particularly evident in Crustaceans [42, 43], which are still largely underrepresented in genomic studies. To date, only 48 out of 727 genome assemblies representing Pancrustacea belong to Crustaceans (with the remaining 679 genomes belonging to Hexapoda) (Genomes-NCBI Datasets, accessed: April 2021). Furthermore, the canonical proPO pathway, considered a core immune response mechanism in the Crustaceans [44], is not represented in the KEGG database. Therefore, we conducted the annotation of the innate immunity related genes in the noble crayfish and the marbled crayfish hepatopancreas transcriptomes using a sequence and domain similarity-based approach. A total of 372 and 353 innate immune-related genes were identified through this approach in the noble crayfish and the marbled crayfish, respectively (Fig. 1, Table S2, Table S3, File S2, File S3).

The identification of these innate immune-related genes provides a basis for future transcriptomic and genomic studies of the innate immunity in freshwater crayfish species. For example, we successfully identified members of the immune signalling Toll pathway. This pathway is conserved in most members of Malacostraca [45] and its activation is critical for antimicrobial peptides (AMPs) expression in Hexapoda [46, 47]. In the noble crayfish and the marbled crayfish, we identified most of the Toll pathway-related genes as single copy (Fig. 1). Recently, an extensive overview of innate immune-related genes has been conducted on numerous marine and freshwater Decapods [45]. The number of TLRs identified in those species ranged between 0 and 8, collocating the number of TLRs found in this study slightly above the higher value (11 in the noble crayfish and 8 in the marbled crayfish)*.* Lastly, in the noble crayfish TOLLIP, Spätzle and Tube were detected in multiple copies (Fig. 1).

The innate immune system in freshwater crayfish is armed with an arsenal of PRRs capable of recognising various PAMPs [48]. The β-(1,3)-glucan receptors (often referred to as Gram-negative binding proteins (GNBPs) or lipopolysaccharide binding proteins) play a vital role in the proPO cascade activation [49]. All GNBPs share a carbohydrate-binding β-glucanase domain as identified in this study [45]. The expansion of this family was previously reported in Decapoda [45], and confirmed in this study with 9 GNBPs identified in the noble crayfish and 8 in the marbled crayfish (Fig. 1). Immune molecules and pathways involved in the response to the *A. astaci* challenge are discussed in detail in the section Molecular mechanisms of the immune response to the A. astaci challenge.

### Gene expression profiles of *A. astaci* challenged crayfish

#### Exploratory analysis of the mapping results

Mean mapping rate of the processed reads for the noble crayfish was 88.96% and for the marbled crayfish 91.98% (Table S4). This was followed by the principal component analysis (PCA), performed to compare the replicates of the *A. astaci* challenged crayfish with the control group. The initial results of the PCA revealed a batch effect in the noble crayfish and the marbled crayfish samples (Fig. S1). For the noble crayfish this effect was related to the differences between male and female individuals, accounting for 21% of the variance. For the marbled crayfish, the highest level of variance (63%) was caused by the differences between reproducing and non-reproducing parthenogenetic females (see Francesconi et al. [41], for details). Therefore, in the down-stream differential gene expression analysis, we accounted for the sex of the noble crayfish, as well as the reproductive status of the marbled crayfish, by including them as factors in the DESeq2 analysis. After batch effect removal, the PCA analysis revealed the grouping only for the *A. astaci* Hap B challenged noble crayfish, while such grouping was revealed neither for other noble crayfish samples nor for the marbled crayfish (Fig. S1).

#### Differentially expressed genes

**Fig. 2:**
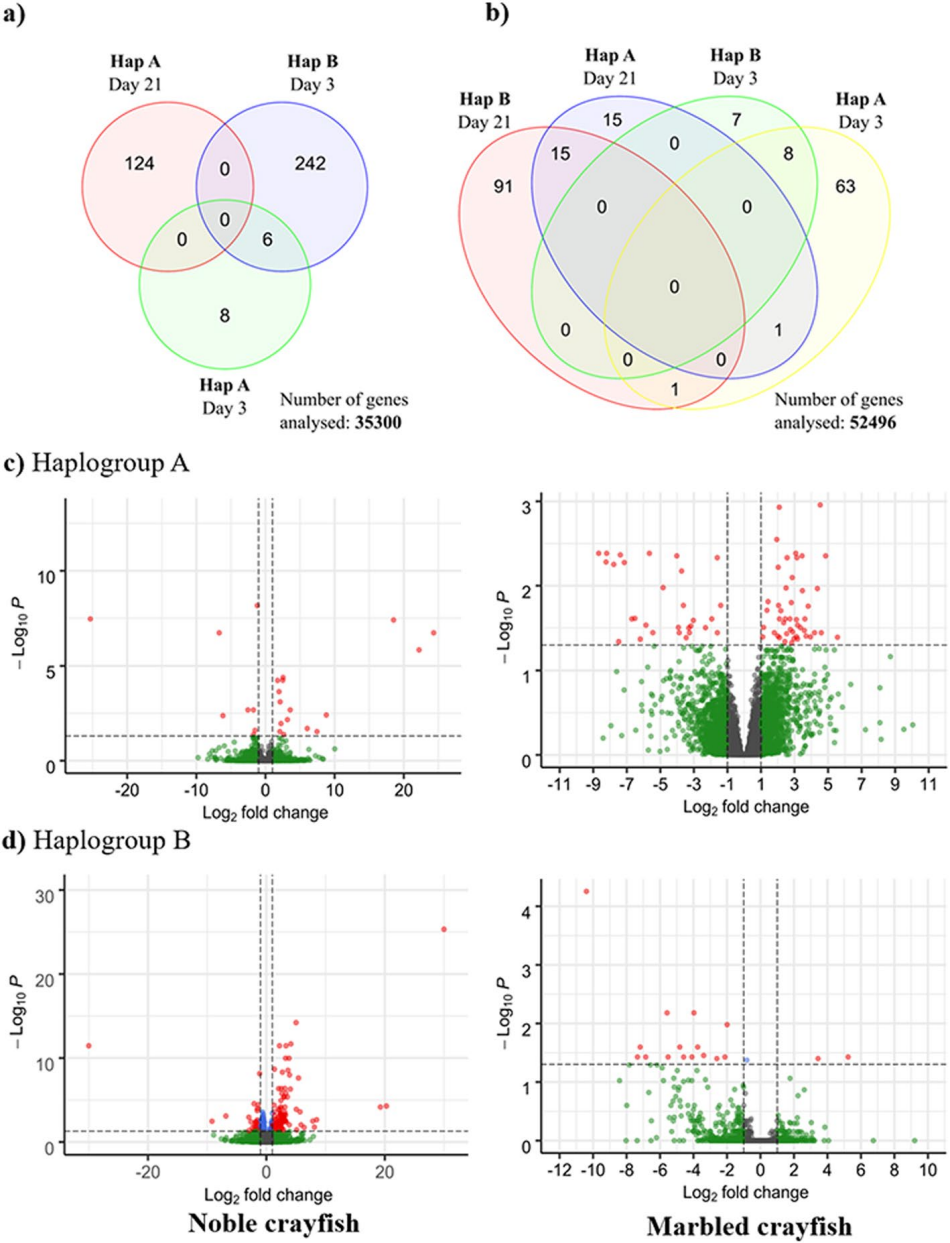
Results of the differential gene expression analysis. (**a**) Venn diagram representing DEGs for all treatments in the noble crayfish (**b**) Venn diagram representing differentially expressed DEGs for all treatments in the marbled crayfish*.* Volcano plots for the noble crayfish and marbled crayfish. **(c)** 3 days post-challenge with haplotype A, (**d**) 3 days post-challenge with haplotype B. The threshold values are represented as dashed lines (*p*-value = 0.05, Fold change = 2). Genes above fold change and *p*-value threshold are coloured red

**Fig. 3:**
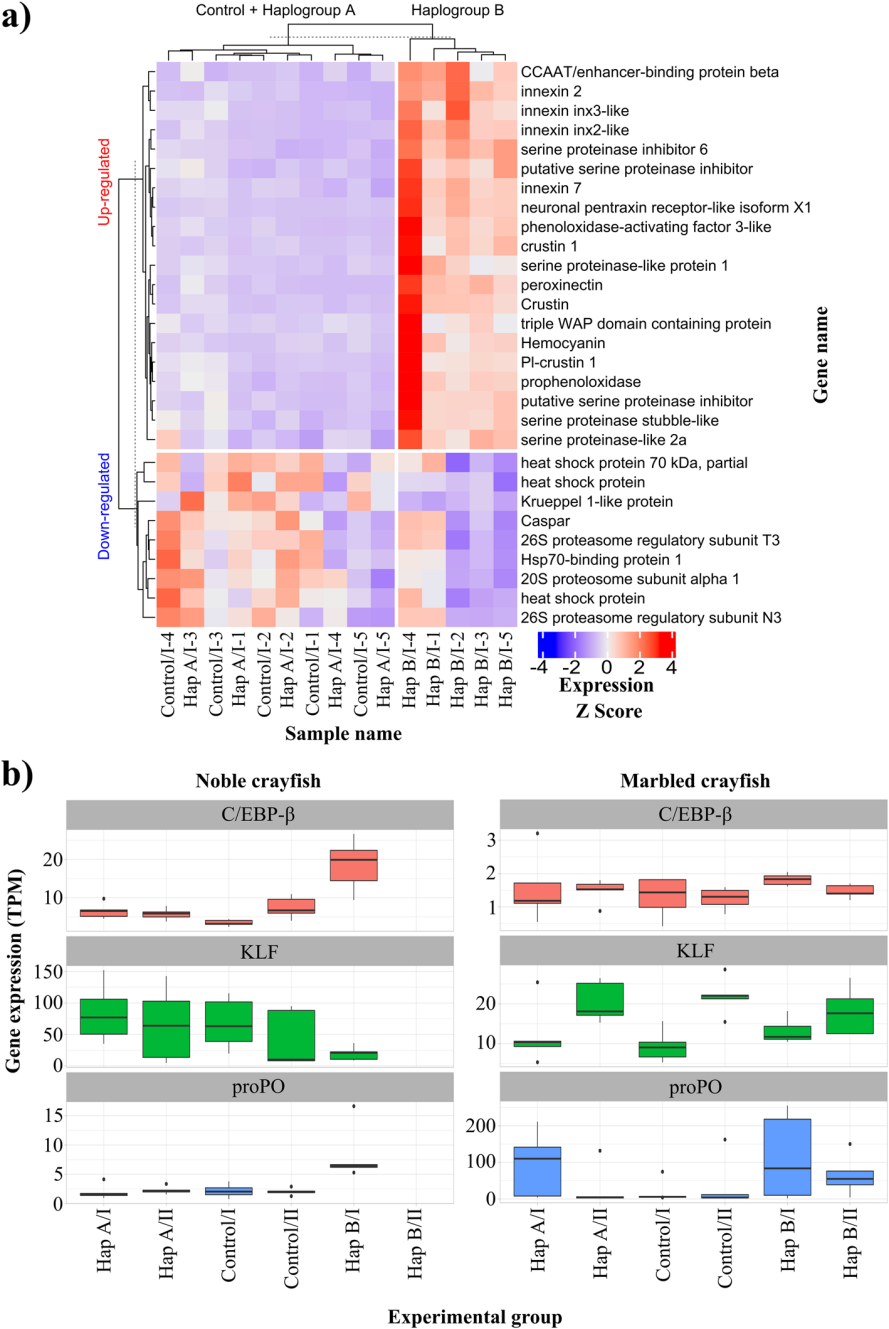
Heatmap of the immunity genes for each sample and treatment detected as differentially expressed in the noble crayfish (**a**) Raw counts were transformed to transcripts per million (TPM), followed by standardisation with Z-score scaling (where Z score is calculated as follows: Z = s_i_-μ/σ where s_i_ is the gene expression for a sample in TPM, μ is mean of the expression for each gene in TPM and σ is standard deviation of the expression for each gene in TPM). Therefore, the colours in the heatmap reflect the relative expression levels between samples per each gene, with higher expression in red and lower expression in blue. Hap A, haplogroup A; Hap B, haplogroup B, I and II, first and second sampling point, respectively (3 days and 21 days post-challenge), 1–5, identifying number of the crayfish (**b**) gene expression of the prophenoloxidase (proPO), CCAAT/enhancer-binding protein beta (EBP), and Krueppel like protein (KLP) in the marbled crayfish and the noble crayfish challenged with *A. astaci*. Expression values are shown in TPM

In the differential gene expression analysis, 35,300 genes for the noble crayfish and 52,491 genes for the marbled crayfish were analysed after removing the genes with low gene counts. In the noble crayfish, a total of 380 DEGs (202 up-regulated and 178 down-regulated) were detected in response to the challenge with *A. astaci* across all treatments (Fig. 2**,** Table S5). The highest number of DEGs was observed in the Hap B challenged noble crayfish 3 days post-challenge, with 243 DEGs (141 up-regulated and 102 down-regulated) (Fig. 2), with many involved in the immune response (Fig. 3). The lowest amount of DEGs was observed in the Hap A challenged noble crayfish 3 days post-challenge, with only 14 DEGs (7 up-regulated and 7 down-regulated) (Fig. 2). The DEGs relevant to the innate immunity, mainly connected to the proPO cascade, were observed in the Hap B challenged noble crayfish 3 days post-challenge (Fig. 2**).** In the marbled crayfish a total of 232 DEGs (102 up-regulated and 130 down-regulated) were detected in the response to the challenge with *A. astaci* across all treatments (Fig. 2, Table S6). The highest number of the DEGs related to the innate immunity was observed in the Hap A challenged marbled crayfish 3 days post-challenge, with 79 DEGs (47 up-regulated and 32 down-regulated), and the highest overall number of the DEGs in the marbled crayfish was observed 21 days post-challenge with the Hap B strain, with 107 DEGs (40 up-regulated and 67 down-regulated). The lowest amount of the DEGs was observed in the Hap B challenged marbled crayfish 3 days post-challenge, with only 15 DEGs, all down-regulated (Fig. 2, Table S6).

Our results indicate the absence of a chronic or a long-term immune response to the challenge with *A. astaci* in both species. The lack of the clear immune response signal 21 days post-challenge suggests that the active immune response in the hepatopancreas had already come to a halt, or was capped below the detection level of the differential gene expression analysis at the time of the second sampling (Enriched gene sets in the response to the *A. astaci* challenge). However, a chronic response could be mediated, as previously suggested in other studies, by circulating haemocytes in the haemolymph of latently infected crayfish [50]. Future studies focused on comparing the gene expression patterns among multiple immune-relevant tissues in the crayfish might clarify this aspect.

#### Enriched gene sets in the response to the *A. astaci* challenge

**Fig. 4:**
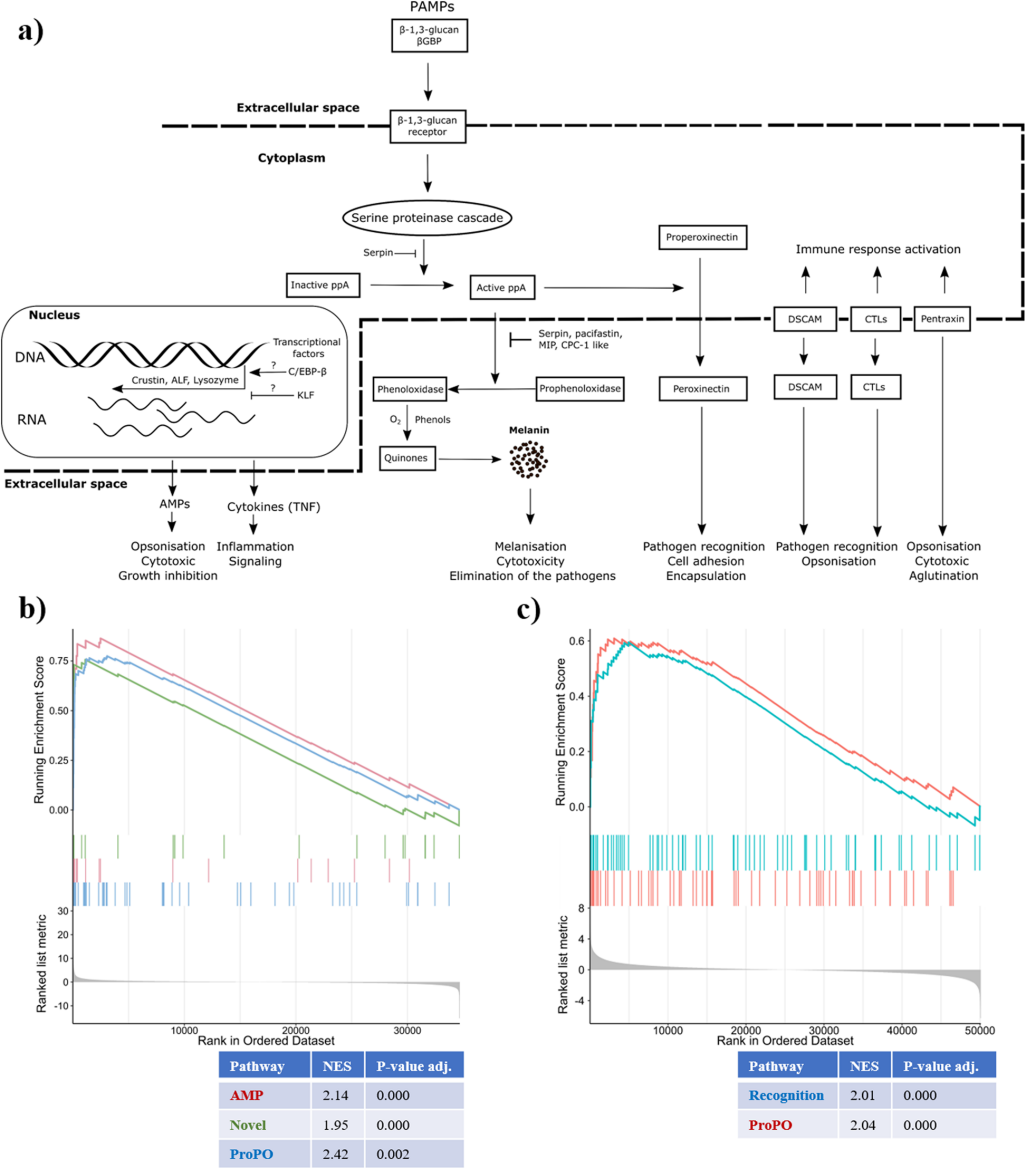
Pathways involved in the freshwater crayfish immune response to *A. astaci* immune challenge, (**a**) Schematic representation of the crayfish immune response to *A. astaci* challenge (**b**) Results of the gene set enrichment analysis for the noble crayfish challenged with Hap B strain of *A. astaci* (Day 3), (**c**) results of the gene set enrichment analysis for the marbled crayfish challenged with Hap A strain of *A. astaci* (Day 3)

As a complementary approach to the differential gene expression analysis, we utilised the newly identified immune-related genes (Immune-related transcripts in the hepatopancreas, the mediator of the crayfish immune response to *A. astaci* challenge) to conduct a gene set enrichment analysis. This approach allowed us to detect moderate or minor changes in the gene expression data [51]. For the noble crayfish, our results revealed the enrichment of AMP, proPO pathway and novel (encompassing novel genes identified in this study) gene sets in the Hap B challenged group (Fig. 4) and recognition gene set in the Hap A challenged group 21 days post-challenge (Fig. S2). The proPO pathway gene set was under-represented in the Hap A challenged noble crayfish 3 days post-challenge. In the marbled crayfish, AMP, proPO and recognition gene sets were enriched for the Hap B challenged group at both sampling points (Fig. S2). Furthermore, in the Hap A challenged group, recognition and proPO gene sets were enriched (Fig. 4). In the marbled crayfish, 21 days post-challenge with Hap A we detected no enriched gene sets. These results, in line with the differential gene expression analysis, suggest that proPO pathway, AMPs and recognition proteins, although not detected as differentially expressed, play a major role in the response to the *A. astaci* challenge. Their interplay and significance are discussed in the text further down.

### Molecular mechanisms of the immune response to the *A. astaci* challenge

#### Activation of prophenoloxidase cascade

Although in both crayfish species the proPO pathway was activated, we detected a substantial difference in the immune response in the two species in the mobilisation of different effector groups and number. The activation of proPO cascade is the most explored humoral response among crustaceans (Fig. 4) [49, 52]. Phenoloxidase (PO), synthesized in its zymogen/inactive form (proPO), is the central enzyme of the pathway. It is cleaved by its activating serine protease (ppA) into the catalytically active PO and the 20 kDA N-terminal fragment (ppA-proPO) with a strong agglutination and bacterial killing capacity [53]. Activated PO is involved in the conversion of phenolic substances into the toxic quinone intermediates involved in the production of melanin, the terminal pathogen encapsulating agent of the proPO cascade [50]. Alongside PO, ppA activates the formation of peroxinectin (PXN), involved in opsonisation, cell adhesion and encapsulation [54, 55]. It was previously assumed, that only the mature haemocytes (granular and semigranular), which are responsible for the release of the proPO in the response to the pathogen stimulation [44, 52], are characterised by the onset of proPO expression [26]. Our results suggest that, alongside haemocytes, hepatopancreas is also involved in the production of the central proteins of this pathway (Fig. 3).

**Fig. 5:**
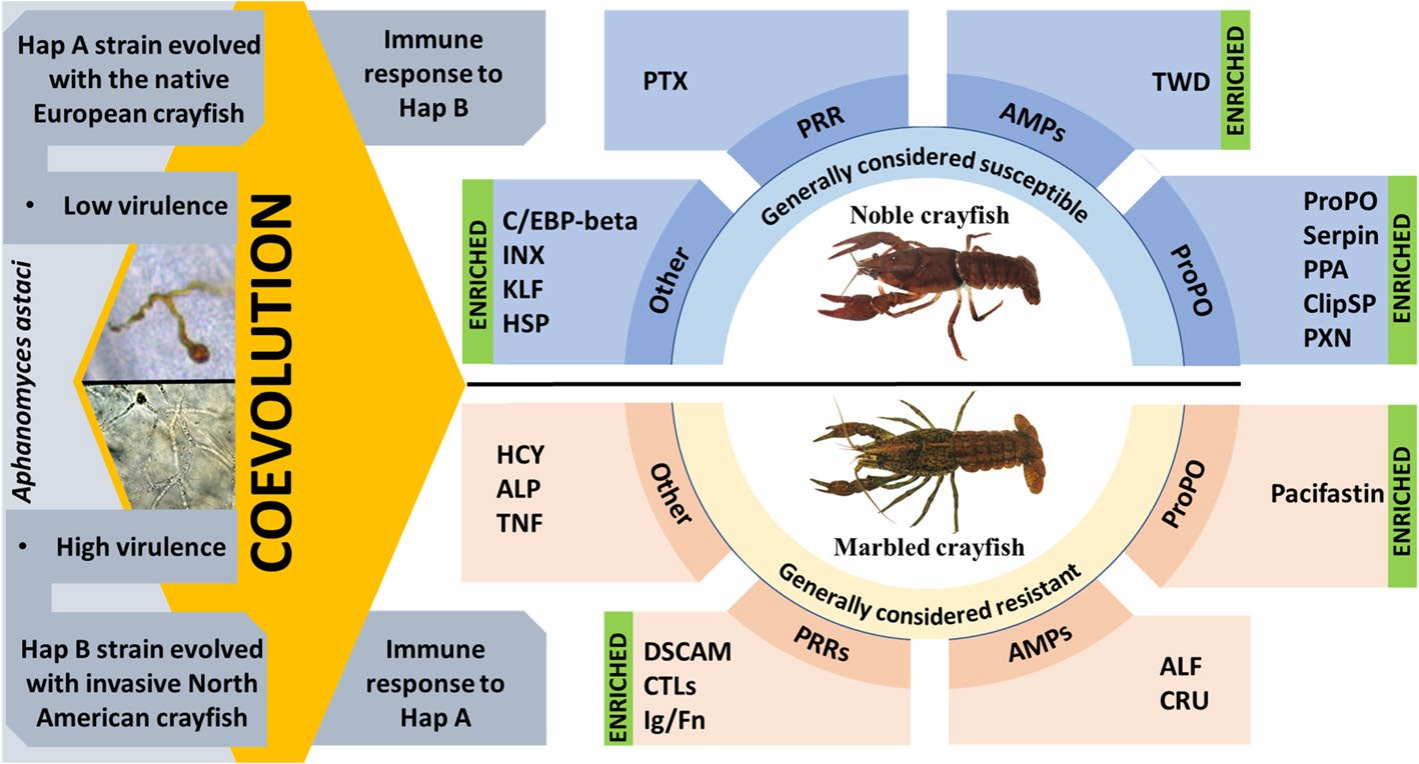
Graphical summary of the experimental results. The noble crayfish and the marbled crayfish were both exposed to two strains of the pathogen *A. astaci*, Hap B of high virulence and Hap A of low virulence. Both species showed immune response to *A. astaci,* although only for one strain. The immune system of the noble crayfish was activated in response to Hap B strain, while the immune system of the marbled crayfish was activated in response to Hap A strain. The utilised Hap A strain has coexisted with European noble crayfish for the past 70 years, and our results indicate that in that time frame it adapted to its new host. On the other hand, the Hap B strain, isolated from its original host in Lake Tahoe, shows a high adaptation to the invasive North American crayfish. Differentially expressed genes (DEGs) were divided in 4 groups: prophenoloxidase cascade related (ProPO), antimicrobial peptides (AMPs), pathogen recognition receptors (PRR) and Other. Enriched gene sets (based on the GSEA) were highlighted. Please refer to abbreviations for the full names of DEGs

In our study we observed an up-regulation of proPO, ppA and peroxinectin in the hepatopancreas of the Hap B challenged noble crayfish (Fig. 3a**,** Fig. 5), while in the marbled crayfish these genes were not differentially expressed in any treatment group. Nonetheless, our findings indicate that the expression of proPO in the hepatopancreas of both susceptible and resistant crayfish can be altered in response to the pathogen stimulation (Fig. 3). In fact, while proPO was not differentially expressed in the marbled crayfish, the variances in the proPO expression levels (transcripts per million, TPM) were much higher in the marbled crayfish challenged with Hap A of *A. astaci* 3 days post-challenge and Hap B of *A. astaci* 3- and 21- days post-challenge, compared to the noble crayfish challenged with Hap B of *A. astaci* (Fig. 3). The results of the GSEA of both treatment groups of the marbled crayfish confirm the activation of the proPO pathway (Fig. 4, Fig. S2). Previous studies detected significant differences between the expression levels of the proPO in the haemocytes of both *A. astaci -*susceptible and -resistant crayfish [24]. Specifically, it was observed that the expression of proPO is continuously elevated in the invasive resistant signal crayfish and the expression levels do not change in response to immune stimuli, while in the susceptible noble crayfish proPO is constitutively expressed at lower levels and its expression levels depend on the presence of the pathogen. The results of our study pointing to a modulation of the expression of proPO in response to the pathogen in the resistant marbled crayfish indicate that the basal expression levels and dynamic of activation of the proPO in the hepatopancreas and the haemocytes are likely different.

Our results indicate that in the Hap B challenged noble crayfish, several serine proteinases (Clip SPs) and serine proteinase inhibitors (serpins) were up-regulated in the response to the infection (Fig. 3, Table S5), and pacifastin-HC gene was up-regulated in the Hap A challenged marbled crayfish 3 days post-challenge (Fig. 5, Table S6). These genes are responsible for the spatial and the temporal control of the proPO cascade (Fig. 4) [50]. Excessive activation of the proPO pathway can cause damage to the host due to the production and the release of toxic quinones, therefore such inhibitory proteins are of utmost importance. In particular, the proteins involved in the proPO regulation are: pacifastin, a regulatory inhibitor of ppA [56]; melanisation inhibition protein (MIP) [57]; caspase 1-like molecule (CPC-1-like), released concomitantly with the proPO and limits the proteolysis of proPO; and mannose-binding lectins [53]. Serpins were reported to play a role in the proPO cascade inhibition [58]. The recognition of the oomycete β-(1,3)-glucan activates the Clip SP cascade responsible for cleavage of the ppA [44]. The up-regulated serpins could also be involved in the inhibition of the oomycete proteinases [59]. Thus, serpins exhibit a dual role as an anti-oomycete agent and as the protectors against the proPO cascade overactivation [33, 60]. This is further supported by the high number of genes encoding for the putative Clip SP (37 in the noble crayfish and 38 in the marbled crayfish) and their inhibitor serpins (19 in the noble crayfish and 24 in the marbled crayfish). The expansion of the Clip SP in Malacostraca (compared to the other Pancrustacea) was previously observed by Lai and Aboobaker [45] with the highest number of the Clip SP (72) observed in the whiteleg shrimp. Co-expression of the proPO cascade effectors and of the proPO inhibitors in the hepatopancreas of the Hap B infected noble crayfish and of the Hap A infected marbled crayfish indicates that the proPO cascade is highly involved in the response to the *A. astaci* challenge. Different elements of the proPO pathway seem to be activated in the marbled crayfish compared to the noble crayfish. Unfortunately, it is not possible to distinguish if this is due to real differences in the expression of the molecules involved in the proPO pathway of the two species, or if it is due to the high individual variance of the responses in the marbled crayfish. Although only one gene was annotated as the putative proPO, multiple hemocyanin (HCY) domain containing genes (14 in the noble crayfish and 20 in the marbled crayfish) were uncovered in both species (Fig. 1). HCY is evolutionarily closely related, but distinct to the proPO [61]. It is believed that Crustacean HCYs can, to a certain extent, mimic the proPO functions [44]. Crustacean HCY is a large type-3 copper containing respiratory protein which forms hexameric structures responsible for oxygen transport [62]. Alongside proPO, in the Hap B challenged noble crayfish, one of the HCY containing proteins was observed as up-regulated (Fig. 3, Table S5). In the marbled crayfish challenged with the Hap A, a highly expressed HCY containing protein was also observed as up-regulated in the hepatopancreas 3 days post-challenge (Fig. 5, Table S6). Unlike vertebrate hemoglobins, HCYs are cell-independent, and are solely suspended in the crayfish haemolymph [62]. This means that the HCYs can be directly excreted from the hepatopancreas, where they are synthesised, to the crayfish haemolymph, without damage to the organism [63, 64]. On the other hand, proPO must be transported to the infection site and incorporated in the granules of semi-granular and granular haemocytes (blood cells) [24, 50]. Shortly after the immune challenge, a significant drop in the number of circulating haemocytes (condition termed haemocytopenia) is observed due to haemocyte mobilisation to the infection site [31, 65]. These haemocytes are mainly directly replaced during haematopoiesis from the hematopoietic tissues [28]. This usually occurs 12–48 hours after the initial challenge [27, 65]. Therefore, during the period of circulating haemocyte depletion, both sensitive and resistant crayfish can rely on the components of the humoral innate immune response, such as antimicrobial peptides and HCYs, until the haemocyte replenishment. This is concordant with the observation by Decker et al., [62] suggesting the innate immunity involvement of the high concentration of HCYs in the circulating haemolymph in tarantula [66]. Finally, HCYs can be proteolytically processed, resulting in a release of AMPs, such as those belonging to the astacidin family [67].

#### Expression of pattern recognition receptors (PRRs)

We observed two up-regulated putative C-type lectins (CTLs) in the marbled crayfish, one in the *A. astaci* Hap A challenged group 3 days post-challenge and one in the *A. astaci* Hap B challenged group 21 days post-challenge (Fig. 5, Table S5). Lectins are a diverse group of proteins capable of binding carbohydrate-binding domains with high specificity [68]. In crustaceans, lectin recognition leads to downstream activation of cellular and humoral responses such as agglutination [69], endocytosis [70], encapsulation and nodule formation [71], synthesis of AMPs [72], antiviral activities [73], and melanisation through the proPO cascade activation [74]. We have identified 55 putative CTLs in the noble crayfish and 43 putative CTLs in the marbled crayfish (Fig. 1). Among PRRs, CTLs have a major role in the innate immunity of freshwater crayfish, where they have also experienced a major increase in their diversity [45].

Among the differentially expressed genes involved in pattern recognition we observed an up-regulated DSCAM 3 days post-challenge in the marbled crayfish challenged with *A. astaci* Hap A (Table S6). DSCAM is a member of the immunoglobulin (Ig) superfamily, with a similar structure in both mammalians and invertebrates. The DSCAM molecule consists of three main components, an extracellular region with several Ig and fibronectin type III domains, a transmembrane domain, and a cytoplasmic tail. Unlike its mammalian counterpart, invertebrate DSCAM exhibits hypervariability in the extracellular domains achieved through a mechanism of alternative splicing during mRNA maturation [75, 76]. In total, we identified 12 putative DSCAM-encoding genes in the noble crayfish and 6 in the marbled crayfish (Fig. 1). DSCAM molecules have been shown to be involved in the antiviral [77] and antibacterial response, mainly in the opsonisation [49]. It is worth noting that due to their hypervariable domain, DSCAMs are considered likely key molecules for the immunological memory in crustaceans [29]. Both CTLs and DSCAMs can exist in a membrane bound and secreted form [78, 79]. Therefore, CTLs and DSCAMs expressed in the hepatopancreas of crayfish can probably be excreted directly to the haemolymph upon the immune challenge, acting as a part of the humoral immune response mechanisms to the pathogen infection.

Alongside the DSCAM we observed another immunoglobulin/fibronectin (Ig/Fn) domain containing protein up-regulated 3 days post-challenge in the marbled crayfish challenged with *A. astaci* Hap A (Table S6). This protein shared 27% identity with the fruit fly (*Drosophila melanogaster*) protein amalgam (Ama, NCBI acc. No.: P15364.2). This amalgam-like protein was 510 amino acid (aa) long, with a molecular weight of 55.63 kDa. It contained 1–21 aa signal peptide domain, three Ig domains (67–158 aa, 166–254 aa, 257–345 aa), and a Fn domain (347–453 aa) with a cytokine receptor motive (439–443 aa). In total, we identified 2 Ig/Fn domain containing proteins with this domain organisation in the noble crayfish and 4 in the marbled crayfish (Fig. 1). The presence of the C-terminal Fn domain clearly distinguishes this protein form the fruit fly Ama [80]. Nonetheless, we can hypothesise that this protein could share the secreted nature of Ama, and its cell adhesion properties [81], potentially having a role in opsonisation, and immune response mediation through its cytokine receptor motive located in the fibronectin domain.

Among the up-regulated DEGs in the Hap B challenged noble crayfish, we identified a pentraxin domain containing gene (Table S5, Pfam: PF00354). The protein product of this gene is 254 aa long (27.95 kDa), with a signal peptide (1–21 aa) on the N-terminus and only 55.5% identity with the neuronal pentraxin receptor-like isoform X2 from the whiteleg shrimp (XP_027224174.1, identified with Blastx). Like the most-well studied pentraxins (e.g. C-reactive protein (CRP) or serum-amyloid P component (SAP)), this pentraxin, due to its size, probably belongs to the group of short pentraxins [82]. We identified 11 putative pentraxin genes in the noble crayfish and 17 in the marbled crayfish (Fig. 1). Pentraxins (or pentaxins) represent a multifunctional and evolutionary conserved group of proteins, with a critical role in the humoral innate immune response [83]. They can recognise a wide range of the pathogen associated molecular patterns, and serve as opsonin, cytotoxic effectors, agglutination promotors or as activators of the complement [82, 84, 85]. Not much is known about the complex system of the complement in the freshwater crayfish and previously hypothesised pentraxin complement activation is most likely not mediated through the C3 component of the complement [84], as it is in vertebrates [85] since C3-like proteins have reportedly been lost in Pancrustacea [45].

In endothermic animals the source of pentraxins is the liver [86] and in the horseshoe crab (*Limulus polyphemus*) and American lobster (*Homarus americanus*) these proteins are produced in hepatopancreas [87, 88]. From there they are released to the haemolymph. Pentraxins are classical acute phase proteins. In humans, the CRP can be utilised as a marker of bacterial and fungal diseases progression [84]. To best of our knowledge, this is the first time a pentraxin-domain containing protein is identified in the crayfish in the response to *A. astaci* infection. This acute protein could be a good indicator of the disease progression. The involvement of the recognition proteins in the response to the *A. astaci* challenge was further supported by the results of the GSEA (Fig. 4, Fig. S2). Application of the acute phase proteins as the markers of the immune status has been previously proposed for the American lobster, where pentraxin-domain containing protein has been recognised as an important component of the immune response to the pathogen challenge [42, 88, 89].

#### Antimicrobial peptides: effectors of the innate immune response

In the noble crayfish challenged with the Hap B strain we identified three up-regulated crustins (Table S5). Among them, of particular interest was the DE triple whey acidic protein (TWP) domain containing crustin, identified in the noble crayfish but with no ortholog in the marbled crayfish. In the noble crayfish we identified 11 and in the marbled crayfish eight putative crustins (Fig. 1). The crustins are part of the cationic antimicrobial peptides AMPs and have three main components: the signal peptide, the multi domain region at the N-terminus and the whey acidic protein (WAP) domain at the C-terminus. They are classified in five groups based on their structure (type I-V) [90]. The crustins are mainly expressed in the crayfish haemocytes, where they can be rapidly secreted directly into the haemolymph during the immune challenge [91, 92]. Some crustins can also exhibit antiprotease activity, possibly inhibiting the proteases secreted by *A. astaci*, limiting the pathogen growth [93]. Recently, a novel TWD containing crustin was described in the red swamp crayfish (*Procambarus clarkii*), showing antibacterial activity [94]. In the marbled crayfish challenged with the Hap B strain we identified one up-regulated crustin 21 days post-challenge (Table S6). The crustins may play an important role in the anti-oomycete response of the freshwater crayfish and require a closer attention in future. The TWD containing crustins might be of special interest, due to their presumed tissue wide expression profiles and participation in the host immunity throughout the whole body [94].

Up-regulated antilipopolysaccharide factor (ALF) was identified in the Hap A challenged marbled crayfish 3 days post-challenge (Table S6), while DE ALFs were not detected in the noble crayfish. This suggests that ALF up-regulation might play a vital role in the resistance of the marbled crayfish towards the *A. astaci* challenge, possibly by binding to the oomycete β-1-3-glucan, hence increasing the host antimicrobial defences acting as an opsonin for the haemocytes [90]. In the noble crayfish, we identified 16 putative ALFs, and in the marbled crayfish we identified 12 putative ALFs (Fig. 1). The ALFs are small proteins with the hydrophobic N-terminal region forming, three β-sheets and three α-helices [45], Pfam: DUF3254. They have been observed in wide range of crustaceans [95], and they are expressed in a wide range of tissues, showing growth inhibiting activity towards bacterial and fungal microorganisms, as well as opsonic activities [96, 97]. Like the crustins, they possess a signal peptide domain and can be excreted [90]. The AMPs were enriched in both the noble crayfish and the marbled crayfish challenged with Hap B strain (Fig. 4, Fig. S2).

#### Innexins: involvement of the gap junction proteins in the crayfish innate immunity

Among the differentially expressed genes, we detected four up-regulated innexins (INXs) 3 days post-challenge in the Hap B challenged noble crayfish (Table S5). These proteins represent the subunits that compose the hemichannel of the gap junctions, and they are analogous to the vertebrate connexin subunits [98].The gap junctions represent the sites of the direct cell to cell communications. This interaction is achieved through the formation of the plasma membrane spanning channels, with each cell contributing to one half of the channel. The mechanisms of gap-junction communications and their repercussions have long been studied in vertebrates, where they are widely distributed across tissues [99, 100]. Although these channels were first observed in the 1950s in the noble crayfish cells, their involvement in the immunity of the freshwater crayfish species is not well understood [101]. We identified 23 putative INXs in the noble crayfish and 20 putative INXs in the marbled crayfish (Fig. 1). For comparison, 8 INXs were identified in the fruit fly, 25 in the roundworm (*Cenorabditis elegans*)*,* 21 in the mediterranean medicinal leech (*Hirudo verbana*) and 6 in the Jonah crab (*Cancer borealis*) [102–105]. In the mud crab (*Scylla paramamosin*), Sp-inx2 expression was up-regulated in the hepatopancreas, the gills and the haemocytes after challenge with bacteria, and was highly expressed in the haemocytes under normal conditions [106]. Although the roles of INXs in invertebrates are largely unknown, based on the current knowledge of the functions of gap junction proteins in other species, we can argue that they could be involved in the antigen processing, as well as in the metabolic and the signalling molecules trafficking [107]. This further establishes the role of the hepatopancreas as a key organ in the distribution of the immune molecules to the crayfish haemolymph [33]. Further studies are needed to elucidate the roles of INXs in invertebrate immunity.

#### Transcriptional factors as novel components in the response to *A. astaci* challenge

Changes in the gene expression levels are controlled through a set of specific transcription factors that interact with the gene regulatory sequences, present in the promoter and enhancer regions. In the Hap B challenged noble crayfish we identified both up-regulated and down-regulated genes 3 days post-challenge, which serve as transcription factors and bona fide play vital roles in the immune response the pathogen (Table S5). One of these genes is a master gene expression regulator belonging to the CCAAT/enhancer-binding protein (C/EBP) family [108]. This family is involved in the regulation of cellular growth, differentiation and death, as well as in haematopoiesis, and immune and inflammatory processes during various diseases [108, 109]. The expression of the putative CCAAT/enhancer-binding protein beta (C/EBP-β), present in single copy in both the noble crayfish and the marbled crayfish, was up-regulated in the noble crayfish challenged with Hap B, while the expression levels in the marbled crayfish remained unchanged (Fig. 1**,** Fig. 3). It has been shown that the expression of the ALFm3 (member of antilipopolysaccharide factor family) in the giant tiger prawn is under the control of C/EBP-β [110]. Previously it has also been shown that C/EBP-β binding sites are present in the crustin Pm7 [111]. The interaction of the C/EBP-β and NF-κB, key transcriptional factor in Toll and IMD pathways was reported during the promotion of the inflammatory mediator’s gene expression [112]. In mice, C/EBP-β is responsible for the control of tumor necrosis factor alpha (TNFα), SAP, complement C3 component expression [108]. This could suggest that the putative C/EBP-β up-regulation is crucial for the acute phase of the *A. astaci* infection in the noble crayfish.

Furthermore, we detected a down-regulation of putative Krüppel 1-like factor protein (KLF1), a member of the Krüppel-like factor (KLF) family, in the noble crayfish challenged with *A. astaci* Hap B (Table S5, Fig. 3). Members of KLF family are transcription factors involved in a variety of metabolic pathways and in the energetic homeostasis of various tissues [113]. KLF1 belongs to a group of KLFs which function primarily as transcriptional activators, although interaction with the transcriptional repressors has also been reported [113]. It is present in single copy in both the noble crayfish and the marbled crayfish (Fig. 1). In the humans, KLF4 is heavily implicated in the regulation of the anti-fungal response to *Aspergillus fumigatus* and *Candida albicans* and was identified as the only transcriptional factor down-regulated during the immune challenge [114]. It has been shown that in whiteleg shrimp (*Litopenaeus vannamei*)*,* the host LvKLF is important for the replication and gene expression of the viral pathogen [115, 116]. In the giant river prawn (*Macrobrachium rosenbergii*), it has been shown that the MrKLF is an important regulator of expression of four antimicrobial peptides, namely Crustin (Crus) 2, Crus8, ALF1, and ALF3 [117]. Knowledge on the expression and the regulation of invertebrates KLF is lacking, therefore conclusive interpretations for the function of the putative KLF1 require further research efforts. Based on the change in the KLF1 expression levels in the noble crayfish, we might speculate that KLF1 repression is important for the activation of the immune response genes in this species. In the marbled crayfish KLF1 expression levels are unchanged during *A. astaci* challenge (Fig. 3).

Together with the KLF1 we also detected down-regulation of the Caspar, a transcriptional suppressor homologous to the Fas-associating factor 1, in the noble crayfish challenged with *A. astaci* Hap B (Table S5, Fig. 3). This transcriptional factor has been shown to play a critical role in the fruit fly, negatively affecting its antibacterial resistance through inhibition of the IMD pathway [118]. In both species the Caspar was detected in a single copy (Fig. 1).

#### Other DEGs in the response to *A. astaci* challenge

Among the up-regulated DEGs in the marbled crayfish we observed several other immune related genes, such as the Tumour necrosis factor (TNF) domain-containing protein (Panther entry: PTHR15151; protein Eiger; putative cytokine) and the lysosomal enzyme putative alkaline phosphatase (AP) (Table S6). The cytokines, class of molecules to which TNFs belong, are heavily involved in the mediation of the immune and the inflammatory responses [119]. They are also known activators of the extracellular trap release (ETosis), a microbicidal mechanism [120]. TNF is also a downstream target of the above mentioned KLFs [114]. Moreover, in the fruit fly, the TNF homolog Eiger is responsible for the release of the proPO in the crystal cells [121]. The TNF is also an activator of the C/EBPβ expression and DNA binding activity [109]. The implication of this gene in the regulation of anti-oomycete responses remains to be experimentally proven in future studies. Alkaline phosphatase, β-glucuronidase, lysozyme, esterases and proteases have been recognised as some of the main lysosomal enzymes in the invertebrates [31]. Lysosomal activity has been implicated in the mechanism of antigen processing in the hepatopancreas epithelial cells and their subsequent release into the haemolymph in the giant tiger prawn [33, 34, 122]. This observation might further establish the role of hepatopancreas in building the immune tolerance to the *A. astaci* challenge.

Interestingly, we uncovered 4 members of the heat-shock protein (HSP) family (HSP70-like, HSP-like-1, HSP-like2 and HSPBP 1) together with proteasome components (20S proteosome subunit alpha 1, 26S proteasome regulatory subunit N3 and 26S proteasome regulatory subunit T3), as down-regulated 3 days post challenge with Hap B strain in the acutely infected noble crayfish (Table S5, Fig. 3). Establishing a correct protein conformation is important for the protein activity. Failure to do so could be due to a lack of molecular chaperons, such as members of the HSP family [123]. Moreover, down-regulation of the ubiquitin mediated proteolysis proteasome genes might have led to the misfolded protein aggregation. It has been shown that HSP 70 is up-regulated in the anti-viral response to the White spot syndrome virus (WSSV) in the giant tiger prawn [124] and the red swamp crayfish [125]. In the fruit fly, it has been shown that the HSP 27 has an antiapoptotic activity, inhibiting the TNF-mediated cell death [126]. This might suggest that during the *A. astaci* challenge, in the acutely infected noble crayfish, a tissue wide apoptosis is in progress.

### Coevolutionary aspects of the host immune response to the pathogen challenge

Our experimental setup, consisting of the noble crayfish and the marbled crayfish challenged with *A. astaci* strains of different origin and virulence, allowed us to make inferences on coevolutionary aspects of the host immune response to the pathogen challenge (Fig. 5). The utilized Hap B strain, characterised by high virulence, was isolated from a latently infected American invasive signal crayfish (*Pacifastacus leniusculus*) host from lake Tahoe (USA). The utilised Hap A strain, characterised by low virulence, was isolated from a repeatedly challenged, latently infected noble crayfish host population, and could have been present in this population for at least 70 years [127]. Consequently, both strains should represent extremes in the mosaic landscape of *A. astaci* strains present in Europe. The results of the infection experiment described in Francesconi et al. [41] showed that the noble crayfish challenged with *A. astaci* Hap B have the highest amount of the pathogen DNA in their tissues, indicating that the pathogen successfully overcame the immune defences of the host. This corresponds to the high number of immune related DEGs observed in this experimental group. Furthermore, it was observed in other experiments (our unpublished experimental results) that all the noble crayfish infected with this specific Hap B strain died within 2 weeks after challenged with the parasite. On the other hand, the Hap A challenged noble crayfish contained the pathogen, without the apparent mobilisation of immune response in the hepatopancreas and were asymptomatic 45 days post-challenge [41]. In the marbled crayfish, the Hap A challenged group showed the highest number of the immune related DEGs, while the Hap B challenged group showed no clear immune response. In fact, in the Hap B challenged marbled crayfish we observed no immune response activation based on the differential gene expression analysis, although enrichment of the proPO, AMPs and recognition gene sets suggesed a low-level mobilization of these pathways (Fig. 4**,** Fig. 5, Fig. S2). Interestingly, the highest amount of pathogen DNA in the marbled crayfish was detected in the Hap B challenged group [41]. This result indicates that the virulence of *A. astaci* and its ability to colonise the host’s tissues are not the only factors influencing the strength of the host’s immune response. In fact, one possible explanation could revolve around processes of coevolution between the crayfish and a specific strain of *A. astaci*.

It has been shown in several instances that invertebrates, although lacking an adaptive immune system, can build an immune memory, mounting an immune response of different magnitude after subsequent exposures to the same pathogen [29, 128]. Such a response could be of tolerance with a lowered immune response to known stimuli, or of potentiation with a higher immune response upon re-encounter of the same pathogen [128]. Furthermore, transgenerational immune priming, in which the immune memory is transferred to the next generations by parents exposed to the pathogen, has been observed in insects [129, 130] and in the brine shrimp (*Artemia franciscana*) [131]. While the specific mechanisms are not completely understood and are likely to be different depending on the host and the parasite, transgenerational immune priming might be the basis of the long-debated host-pathogen coevolution between North American crayfish species and *A. astaci* [14, 132].

It is accepted that coevolution is a dynamic and ongoing process, in which the rapid adaptation of the host to the pathogen (and vice versa) can occur over short time frames, even a few decades [6]. The Hap A strain was isolated from the latently infected noble crayfish in Lake Venesjärvi, Finland. The noble crayfish population in the lake faced at least 3 mass mortalities in the past 50 years until the year 2000. In 2013, the population was identified as carrier of *A. astaci* [127]. The results of our study suggest that, probably in the span of a minimum of 50 years, the Hap A strain used in this study adapted to its naïve native European host, the noble crayfish, presumably through modification of its pathogenic epitopes. This has resulted in the overall lowered virulence of the pathogen. More in general, Hap A contains the first *A. astaci* strains that arrived in Europe likely in 1859s (Alderman 1996). Therefore, it is likely that the prolonged coexistence with other European crayfish species might be leading other strains belonging to this haplogroup through the same adaptation process of the strain used in the experiment.

The noble crayfish utilised in this study come from the population inhabiting Lake Rytky. This population went through a crayfish plague epizootic in the 1980s [19]. Since then, it has recovered and there haven’t been further detections of *A. astaci* presence [19]. The apparent non-activation of the immune system in the noble crayfish infected with Hap A could represent an instance of immune tolerance, in case the *A. astaci* strain that infected the population of Lake Rytky belonged to Hap A. Unfortunately, the haplogroup of that *A. astaci* strain is unknown. Therefore, it is not possible to draw conclusions on how a possible coevolution might have shaped the immune response of the crayfish from Lake Rytky to the *A. astaci* strains tested in the experiment. As Hap A of *A. astaci* adapted to the noble crayfish, the new epitopes presented by this *A. astaci* strain led to the higher expression of the diverse PRR genes in the marbled crayfish, responsible for the recognition of the pathogen and for boosting its immune response capability.

The origin of the marbled crayfish can be traced back to a recent triploidisation event occurred in *Procambarus fallax* from Florida [133, 134]. To date, there are no data on the presence of *A. astaci* in Florida. However, considering the widespread distribution of *A. astaci* in the eastern USA (Martìn-Torrijos et al., 2021) and the elevated resistance of the marbled crayfish to the pathogen (Francesconi et al., 2021), it is likely that *P. fallax* coevolved with some strains of *A. astaci.* The developed resistance to *A. astaci* was then inherited by the marbled crayfish. As *A. astaci* haplogroup B is only distributed in the western part of the USA, it is very unlikely that either of the marbled crayfish or the *P. fallax* encountered strains belonging to Hap B [135]. Yet, the remarkable resistance of the marbled crayfish to the Hap B strain tested in the infection experiment indicates that the presumed coevolution of the *P. fallax* with its native *A. astaci* strain allowed the development of a broad resistance to different strains. Furthermore, in the survey of the distribution of *A. astaci* in the USA, [135] it has been observed that different strains of the pathogen can coexist in the same population and even in the same individual. The elevated diversity of *A. astaci* in its native range and its widespread distribution would create favourable conditions for the selection of the crayfish species with a broad resistance. This is further supported by the lack of *A. astaci* epizootics in North America, even after internal translocation of the freshwater crayfish outside their natural range [135]. Interestingly, the eastern USA is rich in strains belonging to the haplogroup A [135]. If *P. fallax* has been subjected to multiple encounters with strains belonging to this haplogroup, this would further validate our results, indicating that the Hap A strain from Lake Venesjärvi went through quick evolutionary changes to adapt to its new noble crayfish hosts, while become less recognisable for the marbled crayfish.

It has been argued that the harm to the native European crayfish stocks by *A. astaci* would have been much more contained, if the presence of *A. astaci* in Europe resulted only from the first accidental introduction around 1850 [12]. The first mass mortalities would have led to local extinction of the crayfish populations, limiting the spread of the crayfish plague, and potentially causing the disappearance of the pathogen [12]. Unfortunately, the subsequent intentional introductions of different species of North American crayfish, and with them new haplogroups of *A. astaci*, led to an uncontrollable spread of several pathogen strains, which are now firmly established in Europe [12]. While we can conclude that since its introduction into Europe the Hap A strain used in this study went through significant evolutionary changes, the available markers cannot differentiate between this strain and other strains belonging to Hap A, whether present in Europe nor in Northern America [135]. It is increasingly evident that while the genetic markers used until now (RAPD, mtDNA and microsatellites) allow a first general discrimination of the intraspecific diversity of *A. astaci* [21, 136, 137], they are not reliable predictors of the virulence of the strains and of the strains’ potential impact on native European crayfish and thus on freshwater ecosystems. The conservation efforts of native European crayfish would greatly benefit from a genomic approach to analyse the genome-wide intraspecific diversity of *A. astaci.* Such an approach would allow a much finer discrimination between strains, integrating information regarding virulence of the pathogen and its consequences on the freshwater ecosystem. Ultimately, this would lead to better informed and finely tuned conservation actions.

### Study limitations

This study provides a deep insight into the innate immune response following an *A. astaci* challenge in the noble crayfish and the marbled crayfish. Transcriptomic data allowed us to explore the gene expression landscape and to identify key genes in the crayfish immunity. However, information about genomic locations and gene surroundings, which are highly influential on the gene expression profiles, are still not available. Consequently, generating first high-quality genome assemblies for the freshwater crayfish represents a priority in the field of crayfish immunity, and would allow for the future comprehensive epigenomic studies. Unfortunately, until now this has proven to be a challenging task, because freshwater crayfish genomes are often large in size and have a high proportion of repetitive DNA sequences [133, 138, 139]. Furthermore, while in Decapods the role of the hepatopancreas in the immune response against pathogens has already been demonstrated, it has to be considered that the observed expression profile might be influenced by the infiltrating haemocytes [27, 93]. In the future, this issue could be resolved by investigating additional tissues and by applying a higher resolution single cell RNA sequencing, capable of differentiating different cell populations within a tissue [140]. Finally, the gene expression analysis in the marbled crayfish was conducted after removal of the batch effect related to the reproducing crayfish, and this could have biased our results. It has already been shown that immune related genes are over-expressed in the reproducing insects [141]. If, similarly, reproduction in crayfish involves an up-regulation of immune related genes, the removal of the batch effect might have also removed relevant DEGs in the marble crayfish groups. In general, differences in the gene expression caused by different sex, size, age, molt stage and the reproductive status of the experimental animals should be reduced to a minimum by selecting for the experiment animals belonging to the same cohort. This way the possible biases introduced by the removal of batch effects would be avoided.

## Conclusions

Our results indicate that coevolution of the crayfish and a specific strain of *A. astaci* plays a critical role in determining the strength of the host immune response to the pathogen challenge. The lowered virulence of the Hap A strain used in this study and lowered immune response to this strain in the noble crayfish suggest that coevolution between *A. astaci* and the noble crayfish can rather rapidly occur in nature. This host-pathogen co-adaptation raise hope for the future survival of native European crayfish. Nonetheless, repeated introductions of novel *A. astaci* strains represent an overwhelming pressure for the native European crayfish populations, as is evident from the acute response of the noble crayfish to the Hap B strain. Simultaneously, it seems that the ability of the invasive marbled crayfish to mount an adequate immune response to different *A. astaci* strains is much higher, probably due to its North American origin and possible interactions of its closest relative *P. fallax* with multiple *A. astaci* strains. In the light of these results, it is now evident that future research efforts should be aimed towards elucidating the key factors in this active adaptation between the pathogen and the host. Therefore, the identified genes and pathways involved in the immune response to the pathogen *A. astaci* represent a milestone in the conservation and aquaculture efforts for the native European crayfish species. Although our understanding of the freshwater crayfish innate immune response is still limited, it is becoming clearer that multiple organs and a variety of molecular pathways play important roles. Here, we showcased the importance of the hepatopancreas as a highly relevant immune system organ in the response to the *A. astaci* challenge, for both the native noble crayfish and the invasive marbled crayfish. In the immune response of both crayfish species the activation of the proPO pathway was observed. Still, we detected a substantial difference in the immune response in the two species in the mobilisation of different groups and number of effectors. Therefore, it is crucial that future studies are not limited to the analysis of immune response in the haemolymph and to the proPO pathway, but rather consider the multifactorial nature of the innate immune response. Lastly, results provide a basis for the development of the screening assays that will allow detection of the resistant crayfish populations, a promising tool for conservation and management programs.

## Materials and methods

### Aim, design, and study setting

A controlled infection experiment was previously conducted by Francesconi et al. [41] on the marbled crayfish and the noble crayfish. All the crayfish were acclimatised in individual tanks with circulating water for 20 days prior to the start of the experiment. The water conditions (oxygen levels, temperature, conductivity and pH) were monitored daily. The day- night rhythm was mimicked through artificial lights, with 8 h of light and 16 h of dark. All the experimental crayfish were of the similar size, the marbled crayfish (mean carapace length = 39.7 ± 2.7 mm) and noble crayfish (mean carapace length = 43.5 ± 2.3 mm). The crayfish were given every second day preboiled frozen sweet corn. The crayfish were challenged with two different strains of *A. astaci*, a highly virulent Hap B strain and a lowly virulent Hap A strain. All the challenged crayfish were infected with 1000 zoospores per mL. In total 55 individuals (30 marbled crayfish and 25 noble crayfish) were selected for RNA sequencing, with five replicates per treatment (Hap A, Hap B, control) from two time points (3d, 21d post challenge), with exception of the Hap B challenged noble crayfish group, in which all crayfish became moribund in the first days of the challenge and were therefore all sampled in the first time point. For each individual a portion of the hepatopancreas was dissected and snap frozen in liquid nitrogen. Detailed description of the infection experiment and its results Francesconi et al. [41]. The details of the bioinformatical processing of the RNA sequencing reads and transcriptome assembly Boštjančić et al., [142].

### Identification of the crayfish innate immunity genes and taxonomical distribution of transcripts

We retrieved a dataset of innate immunity related genes identified in Malacostraca by Lai and Aboobaker [45]. This dataset was expanded with the selected differentially expressed genes (DEGs) identified in the Hap B challenged noble crayfish. Furthermore, we included the genes specifically related to the proPO cascade. The complete list of the used innate immunity genes and their respective sequences are available in the Table S1 and File S1. Transcriptome assemblies were queried against the subset of the innate immunity related genes with BLASTn and BLASTx 2.10.1+. Hits were then inspected, their function was confirmed based on their e-value (lower than 1e-10), and the presence of the functionally important gene domains identified with a Pfam search.

### Read mapping

All of the sample 2 × 150 bp paried-end reads (Illumina NovaSeq6000; SRA study: SRP318523, read depth: 36.8 M- 68.9 M, mean: 48.59 M) were mapped to the newly obtained reference transcriptome [142] (noble crayfish TSA: GJEB00000000 and marbled crayfish TSA: GJEC00000000) using the pseudo-alignment approach implemented in Salmon 0.13.1 [143]. Several “flags” were used in the Salmon mapping steps to correct the biases that might originate from sequence data: “-validateMappings” [144], “--seqBias” and “--gcBias” [145].

### Differential gene expression analysis

The differential gene expression analysis was conducted according to the DESeq2 protocol [146] implemented in R with the following model design for the noble crayfish: sex (male/female) + groups (Control vs Hap A or Hap B challenge) and for the marbled crayfish: ~reproduction (yes/no) + groups (Control vs Hap A or Hap B challenge). Independent comparisons were conducted for each sampling point. Raw counts from the Salmon output were used as the input. Transcripts highly similar to the marbled crayfish and the noble crayfish mitogenome, respectively, were removed prior to the analysis based on the BLAST hits against the mitogenome (NCBI accession number: KX279347.1 and NC_020021.1). Transcripts assigned to the bacteria and the archaea were also removed based on the DIAMOND search (see **2.2**). results Counts for individual Trinity transcript isoforms were grouped to Trinity genes with the tximport R package [147]. Lowly expressed genes were filtered out: only genes with the raw counts higher/equal to 10 across at least five samples were retained. The package “EnhancedVolcano” [148] was used for the visualisation of the DEGs and “apeglm” for noise removal [149]. The list of DEGs was exported and their counts, log2fold changes and adjusted *p*-values (FDR = 0.1, *p*-value = 0.05) together with their respective annotations were merged. Possible overlaps between the DEGs at different time points were inspected using Venn diagrams [150].

### Gene set enrichment analysis

Enrichment of the innate immunity gene sets identified in the **2.2.** were conducted with ClusterProfiler [151]. Based on the results of the DESeq2 analysis, for each group all genes were ranked according to the following metric: -log10(x)/sign(y), where x is the *p*-value and y log2 fold change. To detect the enriched gene sets we used the GSEA() function, with the *p* values adjusted based on Benjamini-Hochberg correction for the multiple testing (cutoff < 0.01). Graphical representation of the results was obtained using the gseaplot2() function [151].

## Supplementary Information

**Additional file 1: Table S1.** List of sequences used in the BLAST analysis for identification of the innate immunity genes in noble and marbled crayfish and their respective gene accession numbers. **Table S2.** Innate immunity genes identified through the BLAST search with their respective match length, %identity, e- values and Dammit! annotations in the noble crayfish. **Table S3.** Innate immunity genes identified through the BLAST search with their respective match length, %identity, e- values and Dammit! annotations in the marbled crayfish. **Table S4.** Raw and post pre-processing Illumina sequence data statistics and mapping results of the read pseudo-alignment with Salmon against the de novo assembled transcriptome assemblies for noble crayfish and marbled crayfish. **Table S5.** List of differentially expressed genes in the response of the noble crayfish to the challenge with *A. astaci*. **Table S6.** List of differentially expressed genes and their respective annotations in the response of the marbled crayfish to the challenge with *A. astaci*. **Figure S1.** Results of the principal component analysis (PCA) analysis for (a) noble crayfish and (b) marbled crayfish on the rlog transformed datasets, indicating batch effect related to differences between males (blue) and females (red) in noble crayfish and reproduction (reproducing- green, non-reproducing- purple) in marbled crayfish. The PCA with batch effect removal using removeBatchEffect() function implemented in limma R package (Ritchie et al., 2015) for (c) noble crayfish and (d) marbled crayfish. **Figure S2.** Results of the Gene set enrichment analysis for (a) Hap A challenged noble crayfish (Day 3), (b) Hap A challenged noble crayfish (Day 21), (c) Hap B challenged marbled crayfish (Day 3), (d) Hap B challenged marbled crayfish (Day 21). Adjusted *p*- values, and Normalized enrichment scores (NES) are shown. AMPs- antimicrobial peptides, ProPO- prophenoloxidase pathway. **File S1.** FASTA sequences used in the BLAST analysis for identification of the innate immunity genes in noble crayfish and marbled crayfish. **File S2.** FASTA sequences of the innate immunity related transcripts identified through the BLAST analysis in the noble crayfish. **File S3.** FASTA sequences of the innate immunity related transcripts identified through the BLAST analysis in the marbled crayfish.

## Data Availability

All data generated or analysed during this study are included in this article and its supplementary information files. Additional datasets can be found in the co-published data notes article, featuring the required transcriptome assemblies and raw sequencing reads.

## Abbreviations

DSCAM: Down syndrome cell adhesion molecule
proPO: Prophenoloxidase
PAMP: Pathogen associated molecular pattern
PRP: Pattern recognition proteins
TLR: Toll-like receptor
Hap B: Haplogroup B
Hap A: Haplogroup A
DE: Differentially expressed
DEG: Differentially expressed gene
AMP: Antimicrobial peptides
GNBPs: β-(1,3)-glucan receptors
ppA: Serine protease
PXN: Peroxinectin
PO: Phenoloxidase
INX: Innexin
HCY: Hemocyanin
TPM: Transcripts per million
Clip SP: Serine proteinase
Serpin: Serine proteinase inhibitor
MIP: Melanisation inhibition protein
CPC-1-like: Caspase 1-like molecule
CTL: C-type lectin
Ig: Immunoglobulin
Fn: Fibronectin
aa: Amino acid
PTX: Pentraxin
CRP: C-reactive protein
SAP: Serum- amyloid P component
TWD: Triple whey acidic protein
WAP: Whey acidic protein
ALFs: Antilipopolysaccharide factor
C/EBP: CCAAT/enhancer-binding protein
C/EBP-β: CCAAT/enhancer-binding protein beta
KLF1: Krüppel 1-like factor protein
KLF: Krüppel-like factor
s: Crustin
TNFα: Tumor necrosis factor alpha
TNF: Tumour necrosis factor
AP: Alkaline phosphatase
ETosis: Extracellular trap release
HSP: Heat-shock protein
WSSV: White spot syndrome virus
