# Supplementary figures and images for: Host-pathogen coevolution drives innate immune response to Aphanomyces astaci infection in freshwater crayfish: transcriptomic evidence
Source: BMC Genomics. 2022 Aug 22;23:600. doi: 10.1186/s12864-022-08571-z (PMC9394032; doi:10.1186/s12864-022-08571-z)

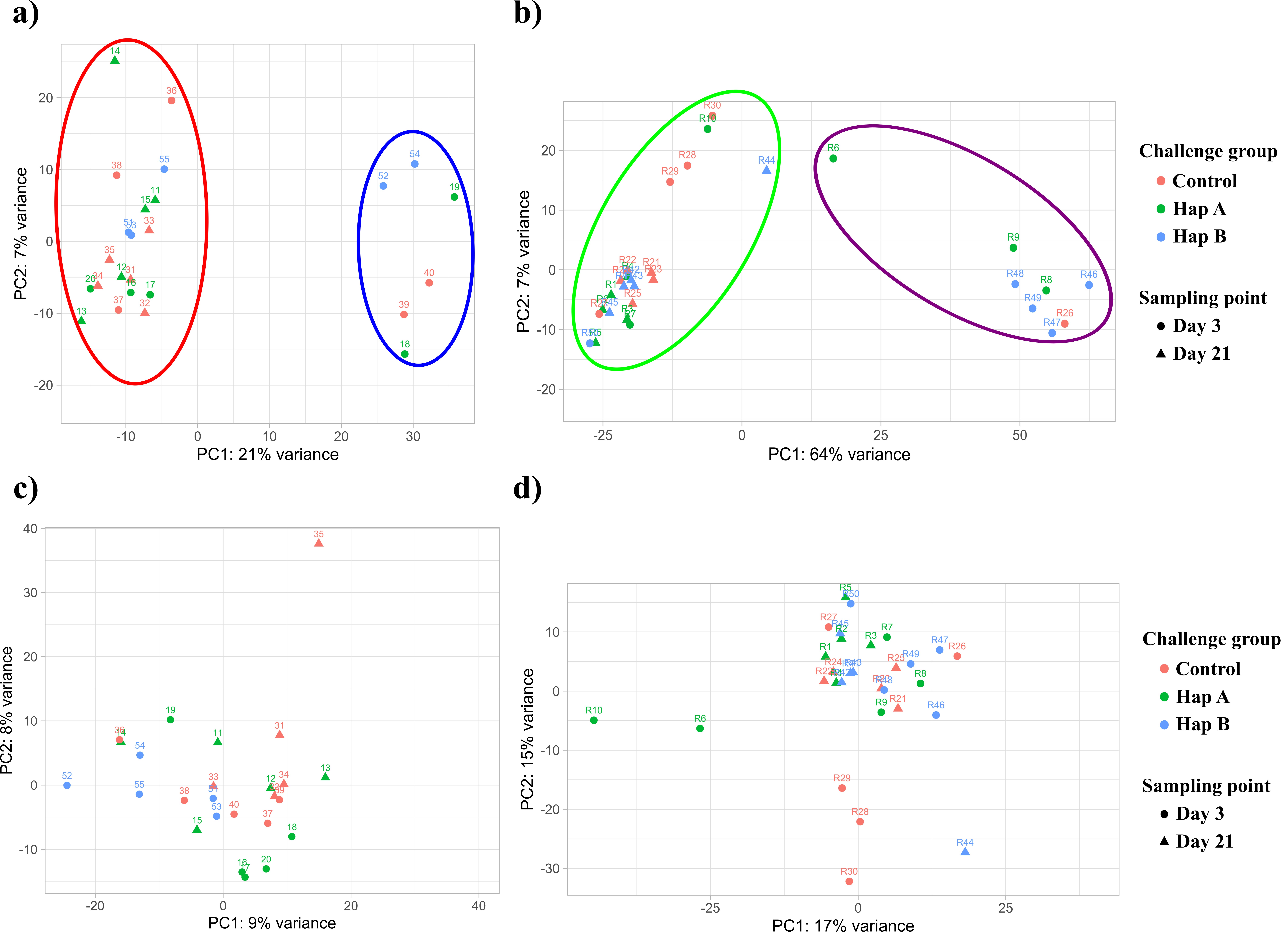

Supplement: Supplementary file 1 — Additional file 1: Table S1. List of sequences used in the BLAST analysis for identification of the innate immunity genes in noble and marbled crayfish and their respective gene accession numbers. Table S2. Innate immunity genes identified through the BLAST search with their respective match length, %identity, e- values and Dammit! annotations in the noble crayfish. Table S3. Innate immunity genes identified through the BLAST search with their respective match length, %identity, e- values and Dammit! annotations in the marbled crayfish. Table S4. Raw and post pre-processing Illumina sequence data statistics and mapping results of the read pseudo-alignment with Salmon against the de novo assembled transcriptome assemblies for noble crayfish and marbled crayfish. Table S5. List of differentially expressed genes in the response of the noble crayfish to the challenge with A. astaci. Table S6. List of differentially expressed genes and their respective annotations in the response of the marbled crayfish to the challenge with A. astaci. Figure S1. Results of the principal component analysis (PCA) analysis for (a) noble crayfish and (b) marbled crayfish on the rlog transformed datasets, indicating batch effect related to differences between males (blue) and females (red) in noble crayfish and reproduction (reproducing- green, non-reproducing- purple) in marbled crayfish. The PCA with batch effect removal using removeBatchEffect() function implemented in limma R package (Ritchie et al., 2015) for (c) noble crayfish and (d) marbled crayfish. Figure S2. Results of the Gene set enrichment analysis for (a) Hap A challenged noble crayfish (Day 3), (b) Hap A challenged noble crayfish (Day 21), (c) Hap B challenged marbled crayfish (Day 3), (d) Hap B challenged marbled crayfish (Day 21). Adjusted p- values, and Normalized enrichment scores (NES) are shown. AMPs- antimicrobial peptides, ProPO- prophenoloxidase pathway. File S1. FASTA sequences used in the BLAST analysis for identif [file 12864_2022_8571_MOESM1_ESM.zip › Supplementary_BMC/Figure S1.jpg]

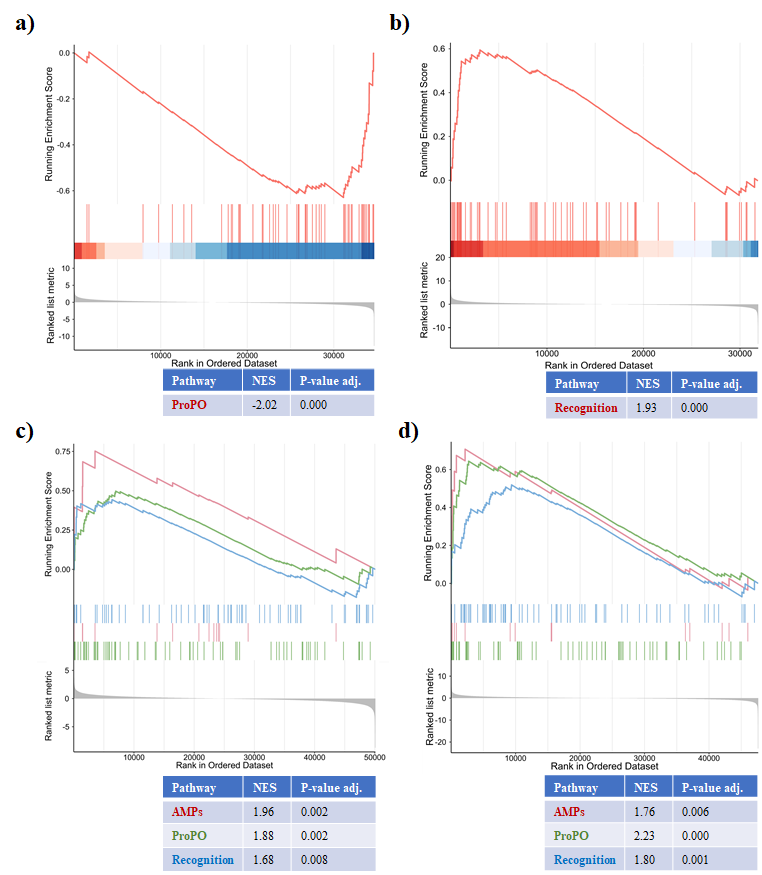

Supplement: Supplementary file 1 — Additional file 1: Table S1. List of sequences used in the BLAST analysis for identification of the innate immunity genes in noble and marbled crayfish and their respective gene accession numbers. Table S2. Innate immunity genes identified through the BLAST search with their respective match length, %identity, e- values and Dammit! annotations in the noble crayfish. Table S3. Innate immunity genes identified through the BLAST search with their respective match length, %identity, e- values and Dammit! annotations in the marbled crayfish. Table S4. Raw and post pre-processing Illumina sequence data statistics and mapping results of the read pseudo-alignment with Salmon against the de novo assembled transcriptome assemblies for noble crayfish and marbled crayfish. Table S5. List of differentially expressed genes in the response of the noble crayfish to the challenge with A. astaci. Table S6. List of differentially expressed genes and their respective annotations in the response of the marbled crayfish to the challenge with A. astaci. Figure S1. Results of the principal component analysis (PCA) analysis for (a) noble crayfish and (b) marbled crayfish on the rlog transformed datasets, indicating batch effect related to differences between males (blue) and females (red) in noble crayfish and reproduction (reproducing- green, non-reproducing- purple) in marbled crayfish. The PCA with batch effect removal using removeBatchEffect() function implemented in limma R package (Ritchie et al., 2015) for (c) noble crayfish and (d) marbled crayfish. Figure S2. Results of the Gene set enrichment analysis for (a) Hap A challenged noble crayfish (Day 3), (b) Hap A challenged noble crayfish (Day 21), (c) Hap B challenged marbled crayfish (Day 3), (d) Hap B challenged marbled crayfish (Day 21). Adjusted p- values, and Normalized enrichment scores (NES) are shown. AMPs- antimicrobial peptides, ProPO- prophenoloxidase pathway. File S1. FASTA sequences used in the BLAST analysis for identif [file 12864_2022_8571_MOESM1_ESM.zip › Supplementary_BMC/Figure S2.tif]
